# Advances in Degradable Embolic Microspheres: A State of the Art Review

**DOI:** 10.3390/jfb9010014

**Published:** 2018-01-26

**Authors:** Jensen Doucet, Lauren Kiri, Kathleen O’Connell, Sharon Kehoe, Robert J. Lewandowski, David M. Liu, Robert J. Abraham, Daniel Boyd

**Affiliations:** 1 School of Biomedical Engineering, Dalhousie University, Halifax, NS B3H 4R2, Canada; doucet.jensen@dal.ca; 2 Department of Applied Oral Sciences, Faculty of Dentistry, 5981 University Avenue, P.O. BOX 15000, Halifax, NS B3H 4R2, Canada; lekiri@gmail.com; 3 ABK Biomedical Inc., Unit 32, 155 Chain Lake Drive, Halifax, NS B3S 1B3, Canada; s.kehoe@abkbiomedical.com; 4 Department of Radiology, Division of Vascular and Interventional Radiology, Fienberg School of Medicine, Northwestern University, Chicago, IL 60611, USA; r-lewandowski@northwestern.edu; 5 Department of Radiology, University of British Columbia, Vancouver, BC V6T 1Z4, Canada; Davidmliu@gmail.com; 6 Interventional Radiology and Diagnostic Imaging Department, Room 3316, Halifax Infirmary Site, QEII Health Sciences Center, 1796 Summer St, Halifax, NS B3H 3A7, Canada; robert.abraham@dal.ca

**Keywords:** resorbable, bioresorbable, degradable, microsphere, embolization

## Abstract

Considerable efforts have been placed on the development of degradable microspheres for use in transarterial embolization indications. Using the guidance of the U.S. Food and Drug Administration (FDA) special controls document for the preclinical evaluation of vascular embolization devices, this review consolidates all relevant data pertaining to novel degradable microsphere technologies for bland embolization into a single reference. This review emphasizes intended use, chemical composition, degradative mechanisms, and pre-clinical safety, efficacy, and performance, while summarizing the key advantages and disadvantages for each degradable technology that is currently under development for transarterial embolization. This review is intended to provide an inclusive reference for clinicians that may facilitate an understanding of clinical and technical concepts related to this field of interventional radiology. For materials scientists, this review highlights innovative devices and current evaluation methodologies (i.e., preclinical models), and is designed to be instructive in the development of innovative/new technologies and evaluation methodologies.

## 1. Introduction

Over the past decade, there has been growing interest in the development of degradable microspheres for transarterial embolization (TAE) procedures; especially for applications in trauma, gastrointestinal bleeding, and for the treatment of uterine leiomyoma. Degradable microspheres are intended to provide effective embolization on a transient basis. Ideally, after achieving their clinical outcome, they are removed from the body without interfering with the functionality of other organs. Unlike conventional permanent agents, degradable microspheres should be designed to optimize the window of therapeutic intent (e.g., embolization). In so doing, these agents may then balance therapeutic requirements, while minimizing the potential of long-term sequelae because of permanent alterations in histological architecture, vascular capacitance and/or injury to both ‘on target’ and ‘off target’ deposition of therapy. A significant driver for the development and utilization of degradable microspheres is that “patients commonly express worries about foreign materials remaining in the body”, and while this may not be a physiological problem, it is certainly an important consideration for patients, and may provide competitive marketing advantages for next generation technologies [1]. 

Although the safety, efficacy, and performance of permanent embolic agents are well established in the clinical literature, degradable microspheres may present new safety concerns. Fortunately, when developing new biomaterials for clinical applications, researchers benefit from the existence of international standards and guidance documents to help address potential risks. With respect to vascular embolization devices, specific guidance documents have been published by regulatory agencies. For example, in 2004 FDA published a document entitled: “Class II Special Controls Guidance Document: Vascular and Neurovascular Embolization Devices”, which lays out special controls for establishing the preclinical safety and efficacy of bland embolic microspheres. This document emphasizes (i) ease of deliverability (from a friction and tortuosity standpoint), (ii) acute complications, (iii) local and systemic foreign body reactions, (iv) recanalization, (v) embolization effectiveness, and (vi) device migration. Given the potential new safety risks that may arise from the use of degradable microspheres, these considerations are critical in the design and evaluation of new microsphere technologies.

Further to such guidance documents, it is also instructive to consider the ideal characteristics of degradable microspheres. These innovative technologies must provide predictable and effective occlusion while also providing:

1. Tailored degradation timeframes—to provide adequate infarction to the target tissues in a variety of indications, subsequently allowing return of flow (e.g., 5–7 h for uterine artery embolization—based on Doppler-guided transvaginal clamping) [2]
2. A variety of tightly calibrated particle size distributions—to optimize particle delivery according to target artery anatomy [3]
3. Ease of delivery through conventional microcatheters—to facilitate adoption of the novel technology into established embolization techniques
4. Full biological compatibility as per the relevant sections of ISO-10993—to minimize safety concerns [4]
5. Multi-modal imageability (e.g., fluoroscopy, CT)—to allow for efficiency and standardization of embolization endpoints [5].

While most of the above points are reasonably self-evident, the last point of multi-modal imageability raises an important and additional design consideration. Specifically, an understanding of the temporal and spatial distribution of embolic microspheres is clinically beneficial [5], with the assurance that degradation byproducts should not, for instance, generate artifacts arising from degradation. 

Prior to developing this article further, readers new to TAE are encouraged to review technical information on techniques and therapies, for example “Transcatheter Embolization and Therapy; Techniques in Interventional Radiology” [3]. It is also important to clarify the definitions and terms utilized in the literature related to degradable microspheres. Terms such as ‘resorbable’ and ‘absorbable’ (with or without the prefix “bio”) are commonly utilized to describe these technologies. However, it must be acknowledged that these terms, which are often used as synonyms for one another, are poorly defined and that despite significant efforts to find consensus about such terms, no agreed consensus in the interventional radiology or broader biomaterials literature exists [6]. Conversely, terms such as ‘degradation’ or ‘degradable’ are scientifically defined throughout the literature. Broadly, degradation refers to “a deleterious change in the chemical structure, physical properties and appearance of materials” [7]. More specifically, and within the context of TAE, degradation may be defined as the cleavage of bonds arising from oxidation, hydrolysis, or enzymatic activity, ultimately culminating in the complete removal of the agent from the human body. Preferably, the degradation mechanism(s) and concomitant byproduct(s) provoke minimal adverse local and systemic responses. For clarity, the remainder of this review will utilize the term ‘degradable’ as per the aforementioned definition.

Based on the special controls described by FDA, as they relate to degradable microspheres for TAE applications, this paper intends to consolidate the highest levels of preclinical evidence relating to the safety, efficacy, and performance of new technologies which are under development as degradable microspheres; specifically, those that are in development for bland embolization procedures. This paper is structured to cross-reference microsphere composition(s) with the special controls provided by the FDA. This format was deliberately chosen to provide a robust framework for discussing the current state of the art technologies with respect to potential risks that may need to be considered as part of a design control process for the development of new degradable microsphere technologies. Finally, a review of the preclinical models utilized by the identified papers will be provided to further highlight the current understanding of the safety, efficacy, and performance of degradable microspheres.

## 2. Methodology

To clearly establish the new materials, which are under development for bland TAE indications, an initial search strategy was completed using search strings with descriptive characteristics for degradable microsphere technologies (e.g., degradable, bioresorbable, bead, microsphere). A summary of the materials identified from this formative analysis is provided in Table 1. Subsequently, each material type was cross-referenced with the peer-reviewed literature using the standard search parameters outlined (Table 1). ‘Web of Science’ and ‘PubMed’ databases acted as primary sources for peer-reviewed literature. Retrieved abstracts were reviewed by Jensen Doucet, Daniel Boyd, Kathleen O’Connell, and Sharon Kehoe.

Eligibility of the papers was established in line with the objectives of this work; specifically, the inclusion criteria adhered strictly to (1) preclinical studies with established control articles (i.e., tris-acryl gelatin, gelatin sponge, PVA), which were (2) directly associated with bland embolization indications and having (3) microspherical morphologies. Papers not meeting these criteria were excluded from the review, along with papers associated with in vitro studies, degradable microspheres for chemoembolization, and opinion-based articles. Included articles are identified and summarized in Table 2. Degradable microspheres intended for use as drug-eluting beads for transarterial embolization have been excluded from this review on the basis that no FDA guidance documents exist with respect to establishing the safety, efficacy, and performance of this type of drug device combinations for TAE. 

## 3. Current State of the Art

Based on the search methods, five materials were identified as candidates for review in this paper. The materials are summarized in Table 2, and comprise: Polylactic-co-glycolic acid (PLGA), PLGA-Polyethylene glycol (PEG)-PLGA, Carboxymethylcellulose-chitosan (CMC-CCN), Chitosan, and Hydroxyethyl acrylate (HEA). Although Chitin was the search term originally entered, its derivative in microsphere form (Chitosan microspheres) warranted inclusion within the assessment, as the chitin agents were all irregular particles. This paper is structured to deal with each of these materials individually based on (1) their basic chemistry as it pertains to their mechanism(s) of degradation, and (2) their respective safety, efficacy, and performance data tabulated against the specific risk mitigation requirements identified by FDA [4]. Further information on the details of the individual parameters regarding safety, efficacy, and performance, can be found in the Class II Special Controls Guidance Document provided by FDA [4]. Finally, the paper provides a brief commentary on preclinical investigation methodologies utilized by those articles included for review.

### 3.1. PLGA

#### 3.1.1. PLGA: Basic Chemistry and Mechanisms of Degradation

PLGA is a hydrophobic, degradable polymer commonly used in drug delivery and medical sutures [8]. It is a linear co-polymer that can be synthesized with different ratios of lactic and glycolic acids [9]. The monomers are linked with an ester bond and depending on the ratio of lactic acid to glycolic acid used in polymerization, different forms of PLGA can be obtained with variable degradation rates [10]. These forms are usually identified based on the ratio of monomers used; for example, PLGA 75:25 identifies a copolymer consisting of 75% lactic acid and 25% glycolic acid [11]. In general, low molecular weight PLGA has been found to degrade more quickly than high molecular weight PLGA, most likely due to its decreased entanglements, allowing water to penetrate the structure more readily and hydrolyze the ester bonds [8]. The degradation of PLGA is well understood and described in detail elsewhere [12,13,14]. Succinctly, PLGA degrades in vivo by hydrolysis of the ester bonds between polylactic acid (PLA) and polyglycolic acid (PGA), yielding PLA and PGA as degradation byproducts [9]. PLA undergoes further hydrolysis to produce monomers, which are metabolized to form lactic acid and then easily excreted through normal cellular activity or converted to glucose to produce adenosine triphosphate [15,16,17]. The degradation of PGA in vivo follows this same process, however the monomer produced is glycolic acid, which is excreted via the kidney or converted to pyruvate for use in the tricarboxylic acid cycle.

#### 3.1.2. PLGA: Safety, Efficacy and Performance

This review identified 1662 articles relating to PLGA based on the search parameters identified in Table 1. Only one of these papers met the inclusion criteria, the remainder of the articles were substantially focused on in vitro studies and materials for chemoembolization. The article that met the inclusion criteria was published by Owen et al. in 2012 [18] and provides a comprehensive and detailed analysis of the safety and efficacy of PLGA-based microspheres for TAE. This paper utilized a uterine artery sheep model over a period of 12 months, with animals divided into four cohorts (1, 3, 6, and 12 months). The control article was EmboSphere^®^ (300–500 µm, Merit Medical Systems Inc., South Jordan, UT, USA). A summary of the article’s findings versus the specific animal testing requirements to establish safety and efficacy as per FDA are provided in Table 3. 

Degradable PLGA microspheres have already been approved by FDA and are available on the market under the brand name Occlusin^®^ 500 Artificial Embolization Device from IMBiotechnologies Ltd. (Edmonton, AB, Canada) [18,19], however human clinical studies have not been published up to the period leading to literature review. To assess the safety, efficacy, and performance of these microspheres, Owen et al. used a particle size distribution of 150–212 µm, comparing this to a conventional product, EmboSphere^®^, with a size range of 300–500 µm in a sheep model [18]. This PLGA particle size range selected by Owen et al. is substantially smaller than that used in most clinical indications, such as hepatic and renal tumor embolization (e.g., 300–500 µm) and uterine fibroid embolization (e.g., 500–700 µm), as well as the control article included in this paper [3,18]. However, it is reasonable to assume this small particle size represents a higher risk with respect to biological response (i.e., higher surface area) and migration, and therefore safety risks in this study were evaluated using approximated worst-case conditions. With regards to delivery, Owen et al. reported it was possible to suspend and visualize the PLGA microspheres in conventional contrast media, and that the microspheres were “easily delivered to target vasculature” using a standard 2.3-French microcatheter without clogging the syringe [18]. The authors note, similar to other published literature that no significant difference in (i) the volume of test and control materials delivered or (ii) the fluoroscopic times required to achieve effective stasis for either product [20].

The PLGA microspheres were shown to degrade in ca. six months, however occlusion persisted up to nine months due to the presence of fibrous ingrowth [18]. Initially, occlusion was mechanical in nature due to aggregation of microspheres within the target vessel. This shifted, over time, to include biological occlusion at one month and three months, as fibrous ingrowth formed “a matrix that held the microspheres in place as they degraded”, maintaining complete occlusion of the treated artery at six months despite the complete degradation of the PLGA microspheres [18]. This is an anticipated biological response given the acidic nature of the degradation byproducts arising from PLGA [21]. By 12 months, normal vessel luminal architecture was observed to be “histologically indistinguishable from the untreated contralateral vessel”, suggesting vessel recanalization (The term ‘recanalization’ is used in a variety of ways in the literature, ranging from re-opening of the occluded vessel to the formation of new vasculature [18]. In this study, the term appears to refer to the reopening of the vessel that has been embolized) [18]. Although PLGA is considered to be a degradable embolic agent in the literature and does technically degrade in vivo, this extended occlusion time may not meet the intended purpose of degradable microspheres or be suitable for the indications proposed for such products (e.g., <24 h for Uterine Arterty Embolization (UAE) [22]). Furthermore, based on its contact type and duration (>30 days), PLGA is technically categorized as a permanent agent according to ISO 10993-1, the international standards used to assess the biological performance of medical devices [23].

Acute complications, such as vessel rupture and perforation, were not assessed in this paper. However, it is reasonable to assume that these complications are not likely a risk associated with PLGA given that it has been cleared by FDA. Further to the generation of fibrous tissue as discussed, both the PLGA and control microspheres were associated with a small number of macrophages and occasional giant cells. Nevertheless, the authors state that “inflammation was not a significant feature of the reaction to either type of microsphere” [18] and no significant systemic foreign body reactions were reported on hematological and clinical chemistry analyses.

Although the risk of migration did not appear to be directly assessed by Owen et al., the authors noted that the control microspheres were detected in non-target vasculature including vaginal, ovarian, and vesicle arteries (63%, 13%, and 6% of the animals, respectively). Conversely, PLGA microspheres were not observed in these structures. This difference may be explained by the compressibility of the control microspheres [24], which likely facilitated passage of the material through small diameter anastomoses joining the uterine artery with the vaginal and ovarian arteries [25]. Furthermore, and perhaps more concerning, was the presence of particles in the vesicle artery, as this was likely a result of reflux out of the uterine artery back into the umbilical artery, resulting in possible non-target embolization [25]. The authors attributed the lack of retrograde flow (reflux to vesicle artery) observed with PLGA to its increased density over the control; it is important to point out that biological occlusion (in the form of fibrotic encapsulation) likely secures the PLGA microspheres at the target level [18]. These observations are of import with respect to designing degradable microspheres. Firstly, inherent to the design, the degradation must be predictable and proceed in a manner that avoids complications associated with non-targeted embolization due passage of smaller particles through the target vascular bed. This may raise safety concerns with respect to the clinical utility of materials designed to degrade in a timeframe shorter than that associated with the development of a sufficient foreign body response (encapsulation of material at the target area), which may mitigate the risk of migration. For example, it is considered in the literature that degradation timeframes of ca. 24 h are sufficient for UAE [2]; however, the host response at this timepoint likely represents transient edema and migration of inflammatory cells without fibrosis. Dichotomously, engineering microspheres that degrade over time periods sufficient to cause biological responses, suitable to mitigating migration risk (i.e., fibrous ingrowth), may contradict the design requirement underpinning the development of degradable microspheres—balancing therapeutic requirements while minimizing collateral damage to adjacent tissue.

#### 3.1.3. Key Advantages of PLGA Microspheres (Occlusin^®^ 500 Artificial Embolization Device)

- Approved by FDA for the treatment of unresectable/inoperable hypervascularized tumors (k093813) [23]
- Available in multiple particle size ranges for a variety of applications
- Easily suspended in conventional contrast media and delivered using standard embolization equipment
- Demonstrated full biological compatibility (via testing performed to obtain device clearance)
- Mitigate the risk of migration through biological occlusion (fibrous ingrowth anchoring the particles in place as they degrade).

#### 3.1.4. Key Limitations of PLGA Microspheres (Occlusin^®^ 500 Artificial Embolization Device)

- Lack of tailorable degradation timeframes—6 to 12 months occlusion timeframe only
- Lacks multi-modal imageability.

### 3.2. PLGA-PEG-PLGA

#### 3.2.1. PLGA-PEG-PLGA: Basic Chemistry and Mechanisms of Degradation

The chemical composition of PLGA and the mechanisms by which it degrades are described in the previous Section 3.1.1. Polyethylene glycols (PEG) are polymers of ethylene oxide with a chemical formula of HO–(CH_2_–CH_2_–O)_n_–H, where n can range from 4 to >400 [26]. From a mechanistic standpoint, the degradation of PLGA-PEG-PLGA begins with hydrolysis of the PLGA crosslinks, yielding PLGA and PEG as the initial degradation byproducts [27]. It is typically regarded that PEG does not degrade, but is excreted unchanged in urine, leading to a limited risk of toxicity [26]. However, should PEG degrade, it is metabolized in the kidney and can be evaluated by the presence of ethylene glycol metabolites, such as calcium oxalate and carbon dioxide, which may pose risk of toxicity [26].

#### 3.2.2. PLGA-PEG-PLGA: Safety, Efficacy and Performance

This review identified 985 articles relating to PLGA-PEG-PLGA based on the keyword search identified in Table 1. Only two of these papers met the inclusion criteria, the remainder of articles were substantially focused on in vitro studies and drug eluting materials. Papers meeting the inclusion criteria were published by Verret et al. in 2014 [2] and Maeda et al. in 2013 [28]. With respect to the former, the study utilized a uterine artery sheep model for a duration of seven days, with tris-acryl gelatin microspheres (500–700 µm) as a control. The latter study used a porcine kidney model for a period of up to seven days with gelatin sponge particles as a control. Summaries of the articles’ findings versus the specific requirements to establish safety and efficacy as per FDA are provided in Table 4. 

The performance, safety, and efficacy of the PLGA-PEG-PLGA microspheres were assessed by Verret et al. using a particle size distribution of 500–700 µm, as it represented the “most common diameter used for uterine fibroid embolization in clinical practice” [2]. Maeda et al. studied this particle size range as well, also incorporating 300–500 µm and 700–900 µm for comparison [28]. No data confirming actual particle size distribution was listed in either study, thus it may be assumed that the size classifications were based on sieve aperture utilized to produce the microspheres. It is worth noting sieve aperture tolerances allow for a degree of error and the actual particle size distributions may be as low as 286 µm and as high as 585 µm for the 300–500 µm range, 480 µm to 815 µm for the 500–700 µm range, and 670 µm to 970 µm for the 700–900 µm range [29]. With respect to injectability and ease of use, no substantial differences between the test and control articles were reported in either study [2,28]. The reported mean volume of particles delivered by both groups showed variability, suggesting different volumes may have been utilized from one animal to next. For example, Verret et al. reported delivering 1.0 ± 0.5 mL of the test article and 1.6 ± 0.9 mL of the control [2]. The volumes of test article delivered by Maeda et al. were notably smaller (0.48 ± 0.17 mL, 0.24 ± 0.11 mL, and 0.24 ± 0.12 mL for the so called ‘REM’ (resorbable embolic microspheres) of 300–500 μm, 500–700 μm, and 700–900 μm respectively), however the volume of control article delivered was comparable to the volume of test article delivered by Verret et al. These discrepancies may be due to variability in animal vasculature—both between and within species but are worthy of note since they may confound the observations. 

PLGA-PEG-PLGA microspheres were reported by both Verret et al. and Maeda et al. to degrade in vitro in PBS in <24 h, and in vivo in less than seven days. Both papers angiographically monitored the animals at three time points, as follows: before delivery of microspheres, 10 min after embolization was achieved, and after seven days. Verret et al. characterized degradation and recanalization at day seven using a three-tier graded system: “normal flow, reduced flow (defined as contrast material visible during five heartbeats before disappearing), and stasis (defined as the blockade of the contrast column in the [uterine artery])” [2]. All animals treated with the PLGA-PEG-PLGA regained ‘normal flow’ by day seven and histological analysis showed no remaining fragments and no arterial wall modifications [2]. Conversely, Maeda et al. reported recanalization as ‘patency rates’, showing it correlated with particle size, as well as level of occlusion (particle distribution), extent of necrosis, and the total percentage of the embolized vessels that recanalized (‘recanalization rate’). It was observed that decreased particle size distributions resulted in more distal occlusion, greater necrosis, and lower recanalization rates [28]. These investigations were conducted with angiography and the authors made strong efforts to correlate them histologically; however, unfortunately, the test microspheres ‘washed out’ into solvent baths during processing, and so the distribution of PLGA-PEG-PLGA could not be directly observed [28]. Accordingly, with respect to Maeda et al., it is difficult to fully evaluate the safety, efficacy, and performance of PLGA-PEG-PLGA microspheres. However, given the encouraging results, future work will likely buttress and substantiate this early data; it would be of benefit to develop methodologies that make it possible to definitively determine the in vivo degradation timeframes of degradable microspheres. Such methodologies would be of immense benefit since it is widely accepted that initial host-material responses, including (but not limited to) protein deposition and cellular interactions, may accelerate or impede the degradation rates of biomaterials [30].

While it may be possible to argue that the degradation byproducts of PLGA-PEG-PLGA (e.g., oxalic acid and its calcium salt) may be a concern with respect to systemic toxicity, evidence for such claims is limited in the literature. Although it is commonly accepted that PEG is excreted unchanged in urine, PEG byproducts can be as large as 20,000 Da, which is significantly larger than the size exclusion of the glomerulus (ca. 7265 Da) [26,31,32]. However, no animals treated with PEG-PLGA-PEG suffered any obvious systemic toxicities, pain, abnormal behavior, or atypical blood counts/biochemistry. With regards to necrosis, Verret et al. found the PLGA-PEG-PLGA microspheres produced significantly less ischemic damage relative to the control (tris-acryl gelatin), attributing this, in part, to the short degradation timeframe of PLGA-PEG-PLGA [2]. Maeda et al. found their control (gelatin sponge) yielded a similar level of necrosis to the smallest PLGA-PEG-PLGA size investigated (300–500 μm), which was significantly higher than the two larger PLGA-PEG-PLGA particle sizes explored. This group did not comment on degradation time as a factor, rather stated the level of necrosis correlated with the distribution of the microspheres, with those positioned distal to the arcuate artery yielding significantly more necrosis. Given the variability in the size distribution of gelatin sponge, it is likely the control agent was present both proximal and distal to this anatomical location, resulting in a higher level of necrosis [28]. 

Neither Verret et al. or Maeda et al. directly investigated the risk of migration of degradable PLGA-PEG-PLGA microspheres (as pointed out by the FDA special control document), and it would be unreasonable to assume that these studies could fully evaluate the multiplicity of risks identified by FDA. Nevertheless, in consideration of the risk, Verret et al. did comment on the absence of observable ovarian damage, concluding migration could not be ruled out solely based on these findings [2]. Given that the predicted degradation timeframe of PLGA-PEG-PLGA is <24 h, embolization would likely be limited to mechanical occlusion (i.e., the material itself) and thrombus formation, without the presence of fibrotic encapsulation (i.e., biological occlusion) to ‘anchor’ the microspheres in places while they degrade. As discussed previously, the absence of this biological occlusion may increase the risk of migration and non-target embolization, as small microsphere fragments may break off and travel forward through small anastomoses, or reflux retrograde into neighboring vasculature [2,28].

#### 3.2.3. Key Advantages of PLGA-PEG-PLGA Microspheres

- Limit necrotic damage due to rapid degradation timeframe (ca. <24 h)
- Available in multiple particle size ranges for a variety of applications
- Comparable ease of delivery to control article.

#### 3.2.4. Key Limitations of PLGA-PEG-PLGA Microspheres

- Lack of tailorable degradation timeframes—ca. seven-day timeframe only
- Toxicity concerns related to PEG degradation byproducts not directly addressed, may not offer full biological compatibility
- Lacks multi-modal imageability.

### 3.3. CMC-CNN

#### 3.3.1. CMC-CNN: Basic Chemistry and Mechanisms of Degradation

Carboxymethylcellulose-chitosan (CMC-CCN) has been proposed as a material for use in TAE based on its ability to rapidly degrade. CMC-CCN polymers can be created with varying degradation times by altering the degree of oxidation of the carboxymethylcellulose (CMC); however, only two time points have been validated in the literature (14 days for 10% oxidated CMC and 30 days for 25% oxidated CMC) [33,34]. In manufacturing, the two components (CMC and chitosan) are combined in a water-in-oil emulsion to form crosslinked polymers via a Schiff base reaction between the aldehyde groups on oxidized-CMC and the amino groups on chitosan [34]. This two-part system avoids small molecular cross-linking agents, which are usually considered to have higher cytotoxic potential [34]. The macrostructure of the resulting polymer is susceptible to degradation by lysozyme, an enzyme that is abundantly present in most parts of the human body; lysozyme hydrolytically cleaves the Schiff base, separating the material into two components (CMC and chitosan) [34].

The first component, CMC, is a non-toxic, biodegradable polymer that is widely used in the pharmaceutical industry [35]. CMC is not degradable by mammalian enzymes but has demonstrated limited in vivo degradation via hydrolysis of its 1,4-glucosidic linkages, producing small amounts of glucose [36,37]. The exact extent of CMC degradation is likely to be determined in future work as the research teams continue to consider the degradation kinetics and compatibility of degradation by-products in future work. 

Chitosan is a naturally occurring polysaccharide derived from the exoskeleton of crustaceans that is commonly used in medicine and pharmaceuticals [38]. It is widely considered non-toxic, having been cleared by FDA for use in wound dressing. Chitosan is not one chemical entity but varies in composition depending on manufacturing; during alkaline hydrolysis of chitin to form chitosan, *N*-deacetylation and depolymerization occur to varying extents. Structurally, chitosan is considered a polymer of glucosamine and *N*-acetylglucosamine, linked by 1,4-glucosidic bonds [39]. Like CMC-CCN, chitosan is also degraded in vivo by lysozyme, which breaks glucosamine-glucosamine, glucosamine-*N*-acetyl-glucosamine, and *N*-acetyl-glucosamine-*N*-acetyl-glucosamine linkages, leaving only glucosamine. Glucosamine then goes on to produce glycosaminoglycans, proteoglycans, and glycolipids in the body. The rate of degradation is historically believed to depend on the acetylation of chitosan, with more acetylated and thus more crystalline chitosans (like chitin) showing faster rates of degradation [38,39,40,41].

#### 3.3.2. CMC-CNN: Safety, Efficacy and Performance

This review identified 417 articles relating to CMC-CCN based on the keyword search identified in Table 1. Only one of these papers met the inclusion criteria, the remainder of articles were substantially focused on drug-carrying materials and neuroprotective effects in ischemic brain injury. The paper meeting the inclusion criteria was published by Weng et al. in 2013 [42] and provides a detailed analysis of the safety and efficacy of CMC-CCN-based microspheres for TAE, which they call ‘bioresorbable microspheres’ (‘BRMS’). This paper used a renal artery rabbit model over a period of 15 min. The animals were divided into three groups: partial occlusion with BRMS-I (CMC-CCN with a theoretical oxidation degree of 10%), total occlusion with BRMS-I, and total occlusion with BRMS-II (CMC-CCN with a theoretical oxidation degree of 25%). Tris-acryl gelatin microspheres (TGMS) with a particle size range of 100–300 µm were included as the control. Angiography was performed before, immediately after, and 15 min after the embolization procedure. A summary of the article’s findings versus the specific requirements to establish safety and efficacy as per FDA are provided in Table 5. 

Beneficially, CMC-CCN microspheres may be produced in a wide variety of particle size distributions ranging from 100 to 1550 µm [34]. For assessment of this technology, Weng et al. utilized a size range of 100–300 µm, providing comprehensive details specific to particle size distribution [42]. This selection of particle size may be justified, similarly to Owen et al. [18], as representing a worst-case scenario in terms of biocompatibility (i.e., surface area) and risk of migration (Section 3.1.2). Importantly, and with regards to ease of use, Weng et al. stated the injection of CMC-CCN microspheres was “easily performed without any clogging or clumping” using a 2.8-F microcatheter [42]. The control article was deemed significantly more difficult to use as it tended to stick to both the syringe and the microcatheter, substantially reducing the percentage of particles delivered per syringe. The authors attributed the decreased ‘stickiness’ observed for the CMC-CCN microspheres to the low coefficient of surface friction inherent to hydrogels [42]. 

Degradation rates of CMC-CCN microspheres have been previously demonstrated in vitro and depend on the level of oxidation from processing [43]. In the present study, the timeframe of 15 min did not allow for assessment of in vivo degradation or recanalization. Nonetheless from a performance standpoint, the authors stated that more cross-linking can provide for a slower rate of degradation [42,43]. The key focus of this paper was related to the acute phase of embolization, focusing on initial particle distribution and level of occlusion. Comparisons regarding material performance were made when total occlusion (effective stasis) was used as an end point (versus a pre-determined dose). Interestingly, microsphere distribution was reported as being dependent on the level of CMC-CCN crosslinking. Weng et al. found BRMS-II microspheres (oxidation degree of 25%) occluded slightly more proximal than BRMS-I (oxidation degree of 10%) and the control (which was equal to BRMS-I), likely due to their lower compressibility. Despite these observations, the authors reported no statistical differences in the diameter of occluded vessels or the magnitude of particle deformation between any of the materials [42]. The CMC-CCN microspheres remained intact in all histological specimens; however, 20% of the control particles showed pitting or ‘peeling’. The authors stated this may have been a result of the histochemical processing and agree that it may have obscured their analysis of microsphere deformation [42].

Given the focused and acute scope of this study, it was not possible to comment on acute complications and foreign body reactions. On histological evaluation, the authors discussed the presence of ‘white spaces’ surrounding the microspheres, attributing them to either “dehydration of the microspheres and shrinking during the staining process, or slicing that was not through the cross-section of the vessel diameter” [42]. It may also be possible these spaces resulted from thrombus formation, as the authors noted “visible tissue and blood cells that surrounded the microspheres were erased” prior to analysis [42]. Although risk assessment was limited in this paper due to its short timeframe, the authors have published a subsequent study, which is considerably more focused on the special controls published by FDA; this follow-up study was not included in the present review as it did not meet the inclusion criteria (lack of commercial control), but is cited so as to direct the readers toward additional data for these materials [44].

#### 3.3.3. Key Advantages of CMC-CNN Microspheres

- Potentially offers a range of tailorable degradation timeframes based on in vitro evaluations
- Available in a wide variety of particle sizes from 100 to 1550 µm
- Superior ease of delivery as compared to the control (less adhesive) using conventional embolization equipment.

#### 3.3.4. Key Limitations of CMC-CNN Microspheres

- No information on in vivo degradation timeframes or recanalization
- Toxicity concerns related to degradation byproducts and their size(s) not addressed, may not offer full biological compatibility
- Lacks multi-modal imageability.

### 3.4. Chitosan

#### 3.4.1. Chitosan: Basic Chemistry and Mechanisms of Degradation

Chitin is a biopolymer found in the shells and exoskeletons of animals, such as insects, squids, and crustaceans, and serves a similar structural role to that of cellulose in plants or collagen in higher species [45,46]. Structurally, chitin has a highly-extended hydrogen bonded semi-crystalline arrangement, which limits its solubility in water [45]. The chemical composition of chitin is poly(β-(1-4)-*N*-acetyl-d-glucosamine). Chitosan is the *N*-deacetlyated derivative of chitin and contains multiple free amino groups, typically with a degree of deacetylation > 0.65 (deacetylation values of 75% and 99% were found in the specific body of literature explored for this paper) [47]. The degradation mechanism and products of chitosan have already been addressed in the section on CMC-CCN (Section 3.3.1). 

#### 3.4.2. Chitosan: Safety, Efficacy and Performance

This review identified 585 articles relating to chitin and chitosan based on the keyword search identified in Table 1. Only 1 of these papers met the inclusion criteria, the remainder of the articles were substantially focused on in vitro studies and drug-loaded materials. The paper meeting the inclusion criteria was published by Kwak et al. in 2005 [47] and provides a comprehensive and detailed analysis of the safety and efficacy of chitin- and chitosan-based particles for TAE. While this paper examined many morphologies (e.g., thin, scale-shaped chitin and chitosan plates), the current review focuses solely on data relating to the chitosan microspheres (75% deacetylated). This paper used a renal artery rabbit model over a period of 32 weeks. The animals were divided into nine cohorts: 1 and 3 days, and 1, 2, 4, 8, 16, 24, and 32 weeks, and PVA particles (150–250 μm) were used as the control. A summary of the article’s findings versus the specific requirements to establish safety and efficacy as per FDA are provided in Table 6. 

To assess the safety, efficacy, and performance of chitosan microspheres, a uniform size distribution of 150–250 µm was used for all test and control agents. The authors provided detailed particle size distributions, reporting the mean ± standard deviation of chitosan microspheres as 271 ± 37.2 µm and non-spherical PVA particles as 326 ± 89.1 µm [47]. The notable increase in mean PVA particle size as compared to the labelled size range can likely be attributed to the rod-shaped morphology of this material, which enabled passage through 150–250 µm sieves. Spherical agents were not included for control purposes, as the authors reported they were “not available at the time [their] study was performed”. With regards to ease of use, the authors reported both the chitosan microspheres and control were “easily injected through the catheter without causing any blockage” [47]. The distinct morphologies of the two materials lead to varied degrees of occlusion; the chitosan microspheres filled the vessel lumen more compactly than the irregular PVA particles. The authors do not provide the volume of material delivered per injection.

Chitosan microspheres were found to have a degradation timeframe of ca. 32 weeks. The first signs of degradation appeared at 24 weeks, with complete absence of the microspheres at 32 weeks [47]. Although PVA is known to be non-degradable and is therefore regarded as a permanent agent in the literature, this group found the size and number of PVA particles decreased over time. This began after eight weeks and persisted for the duration of the study. Though unlike the chitosan microspheres, PVA particles were still present in situ after 32 weeks. The authors suggested this observation, along with the presence of PVA fragmentation and foreign body giant cell infiltration, may indicate PVA underwent some sort of degradation process(es) [47]. Recanalization (reopening of occluded blood vessels) was not explicitly studied in this paper, although the authors did comment on the formation of new capillary growth that correlated with the degree of fibrosis [47]. This was present for both chitosan microspheres and PVA particles, though it occurred earlier in the PVA cohort (week two vs. week four) and to a more substantial degree. The presence of fibrosis, in combination with the extended amount of time both embolic agents were present in the vessels, suggest there was a very good chance that particle degradation did not lead to vessel reperfusion in either case [47]. This extended occlusion time lends itself to the same argument as noted above for PLGA (Section 3.1.2); although both materials may eventually degrade in situ, both chitosan and PVA can essentially be categorized as permanent embolic materials due to their protracted biological occlusion times [23].

Acute complications were discussed effectively in this paper, owing to the use of two additional stains, Victoria blue and Masson trichrome, allowing for thorough examination of elastic lamina damage and fibrosis, respectively. Victoria blue staining demonstrated that vascular injuries became more severe over time in both groups, progressing from damage of only internal elastic lamina to damage of the middle membrane. There was, however, no hemorrhage or extravasation of either material. The authors proposed that the destruction of the elastic lamina occurred because of either “ischemia, direct toxic effects of embolized materials, or focal angionecrosis by embolized materials to the vessel wall” [47]. Inflammation was seen in relation to both particle types to varying degrees, beginning immediately after embolization, and persisting up to roughly one week. The inflammation observed with chitosan microspheres, although more severe than that of the control, was not severe enough to destroy the vessel wall. After approximately one-week, inflammatory infiltrates aggregated into foreign body giant cells, which persisted up to week 32 for both materials; these foreign body giant cells presented before degradation of the material and persisted continuously after the chitosan microspheres were completely degraded [47]. With regards to systemic reactivity, neither material altered blood chemistry. Specifically, the authors emphasized the importance of the low eosinophil count, which suggests chitosan microspheres are unlikely to evoke allergic reactions [47].

Although not formally assessed, the issue of migration arose due to complications seen in this study. One rabbit died following reflux of chitin particles (irregular particles not discussed in detail in this review) from the right renal artery to the left, resulting in occlusion of both renal arteries. The authors discussed this complication in relation to the volume of material, stating this occurred when an excessive number of particles were injected. Unfortunately, the volumes of each material delivered were not provided [47] and the issue of reflux was not discussed any further or specifically in relation to material properties, such as embolic morphology or density. As previously mentioned, the extended timeframe of chitosan microsphere degradation likely mitigates, to a degree, the risk of embolic migration, as fibrous ingrowth anchors the microspheres in place as they degrade. Nonetheless, this theory merits proper exploration prior to use in human subjects. 

#### 3.4.3. Key Advantages of Chitosan Microspheres

- Easily delivered through conventional microcatheters and provides for more compact vessel occlusion (relative to irregular PVA particles)
- Low risk of local and systemic toxicity (unlikely a high-risk allergen)—potential to fulfill full biological compatibility
- Extended degradation timeframe potentially mitigates risk of migration through stimulation of fibrous ingrowth.

#### 3.4.4. Key Limitations of Chitosan Microspheres

- No information provided on ability to manufacture different particle size ranges
- Lack of tailorable degradation timeframes—24 to 34 week occlusion timeframe only
- Lacks multi-modal imageability.

### 3.5. HEA

#### 3.5.1. HEA: Basic Chemistry and Mechanisms of Degradation

Hydroxyethyl acrylate (HEA) is a hydrolytically degradable material that has been proposed for embolization procedures due to its resiliency and lack of dependence on enzymatic degradation [48]. HEA is synthesized to contain two degradation sites using a *N*,*N*′-(dimethacryloyloxy)adipamide crosslinker (C_6_NCL) to modulate the rate of degradation while maintaining desired mechanical properties. HEA undergoes degradation in basic conditions, which is an important consideration when designing HEA microspheres, since embolization procedures are intended to produce ischemic events (resulting in acidic conditions) and cessation of flow (preventing elimination of carbon dioxide) [49]. The degradation of hydroxylamines substituted into the material, such as C_6_NCL, is known to lead to production of primary amines (e.g., putrescine) and linear carboxylic acids with the loss of carbon dioxide [48]. Although these degradation byproducts (e.g., putrescine) are often naturally present in the body, they may be toxic in large quantities [50].

#### 3.5.2. HEA: Safety, Efficacy and Performance

This review identified 65 articles relating to HEA based on the keyword search identified in Table 1. Only one of these papers met the inclusion criteria, the remainder of articles were substantially focused on in vitro testing and studies related to high environmental ammonia. The paper meeting the inclusion criteria was published by Schwarz et al. in 2003 [48]. This paper used both renal artery canine and auricular artery rabbit models over a period of three weeks, with EmboGold microspheres (300–500 μm, Merit Medical Systems Inc., South Jordan, UT, USA) as the control. A summary of the article’s findings versus the specific requirements to establish safety and efficacy as per FDA are provided in Table 7. 

The safety, efficacy, and performance of HEA microspheres were evaluated by Schwarz et al. using a particle size range of 300–500 µm, which was obtained via sieving [48]. The authors did not provide any commentary regarding the ease of use of either product, and no values for average volume delivered per injection were provided. It was, however, mentioned that the transparency of HEA microspheres rendered them practically invisible in a syringe. To mitigate this issue and facilitate easier handling of this product, HEA microspheres were colored orange using small amounts of fluorescein acrylate [48].

Schwarz et al. began their study by investigating the degradation rate of synthesized HEA microspheres in vitro (pH 7.4, 37 °C), demonstrating complete degradation of the material at 22 days. This correlated, to a degree, with what was observed in their in vivo investigation; on angiography, embolized vessels were recanalized at three weeks. However, histopathology showed HEA microspheres were often present in situ at three weeks in “various stages of degradation and phagocytosis”, indicating (i) that degradation was incomplete at this time point (ii) highlighting potential discrepancies between in vivo and in vitro degradation findings [48]. In areas where the HEA microspheres had completely degraded, residual inflammation and neointimal thickening were observed. As discussed by Shwarz et al., this discrepancy between in vitro and in vivo degradation timeframes may be a result of the acidic conditions produced during ischemic events and cessation of blood flow [48]. Nevertheless, the authors effectively showed using both renal and auricular models that temporary arterial occlusion could be achieved using HEA microspheres for up to two weeks [48]. 

Risks associated with acute complications and systemic toxicity were not reported for HEA microspheres in this work. Little information was provided regarding foreign body reactions other than noting that both HEA microspheres and the control lead to inflammation and tissue infarction, with the permanent agent demonstrating notably higher levels of both. In line with this, the authors stated, “the theoretical advantages of temporary embolization were not convincingly demonstrated in [their] model since temporary renal branch occlusions for the time periods investigated still led to tissue infarction” [48]. It should be noted that a renal artery model is an end vessel model and may not be ideal for correlating infarction and degradation. The ideal degradation timeframe for embolic microspheres remains unclear and likely varies across different clinical indications. Although HEA yielded ischemic insult to the healthy tissues examined by Schwarz et al., this may not have occurred, to the same degree, in the presence of a tumor/fibroid. Due to the hypervascular nature of tumors/fibroids, these diseased tissues have been shown to preferentially attract blood flow and therefore microspheres, sparing neighboring healthy tissues. Consequently, the ischemia observed by Schwarz et al. may not be representative of a true clinical scenario. HEA microspheres reliably degraded, succeeding in providing patients with a dependable material that will be eliminated from their body—a key driver in the development of degradable microspheres for TAE.

The risk of microsphere reflux was briefly mentioned in the methods of this paper as a potential complication to avoid; however, it was not discussed in detail, as unlike Kwak et al. (Chitosan, Section 3.4), no animals died from microsphere migration. The authors alluded to microsphere encapsulation in some tissue samples, but it appears this was not always the case. As such, it is unclear, and perhaps unlikely, a biological mechanism would be reliably in place to anchor HEA microspheres during degradation, suggesting migration would be possible.

#### 3.5.3. Key Advantages of HEA Microspheres

- Produced notably lower levels of ischemia relative to control agent

#### 3.5.4. Key Limitations of HEA Microspheres

- No information provided on ability to manufacture different particle size ranges
- No information provided on ease of use of microspheres
- Lack of tailorable degradation timeframes—2 to 3 week occlusion timeframe only
- Toxicity of degradation byproducts (e.g., putrescine) not assessed
- Lacks multi-modal imageability.

## 4. Preclinical Models

Selecting an appropriate model for the preclinical evaluation of embolization products is critical for accurately assessing safety, efficacy, and performance prior to human use. While some parameters can be assessed in vitro (e.g., particle size and ease of use), animal models are required to best evaluate the material in an environment that approximates the intended clinical scenario [51]. Specifically, with regards to the list of special controls provided by FDA, in vivo models are required to assess degradation and recanalization, host response (acute complications and local and systemic reactions), and migration [11]. As no animal model can truly represent the intended clinical conditions in their entirety, it is necessary to match the specific objectives under consideration to the best available model. Unfortunately, this proves to be a complicated process, as several variables are at play that can significantly alter outcomes and distort results – anatomy, vascular distribution, blood flow, and immune and coagulation responses are examples of such [51]. The selected animal, organ, and tissue condition (e.g., tumor vs. healthy) are three key factors that affect these variables, which must be carefully selected to meet a study’s objectives [52]. The following section is intended to outline the advantages and disadvantages of the models incorporated in the papers considered in this review.

The kidney and uterus were the two main organs used to carry out the preclinical analyses reviewed in this paper. Embolization procedures are performed clinically in both of these organ structures (e.g., for renal cell carcinoma, uterine fibroids), and thus these models are clinically relevant. Both of these organs act as a filter, in which microspheres may be delivered, and entrapped, as the vascular diameters decrease from the renal/uterine artery down to end arterioles [53]. The uterus is perhaps less straightforward, in that the uterine artery often anastomoses with the contralateral uterine artery, ovarian artery, and/or vaginal artery [53]. In theory, these structures are suitable for evaluating the migration risk associated with degradation, as they allow the material to advance deeper into the vascular bed as it decreases in size, but remain entrapped within the organ (i.e., not shunt elsewhere). In the papers under review, the kidney and uterine models enabled, for the most part, effective evaluation of degradation, recanalization, and host response; however, commentary regarding migration was limited. 

Microsphere migration is perhaps the most difficult parameter to investigate, as vascular patterns and blood flow are so variable in different species, organs, and disease states [52]. None of the papers discussed in this review considered a diseased model, and thus the vascular distribution and blood flow dynamics limited the applicability of these results to clinical use for several reasons. Firstly, vascular tumors (including uterine fibroids) tend to show significant increases in blood flow relative to their non-diseased counterparts, and preferentially draw microspheres from the circulation to the tumor [54]. Secondly, the vasculature of tumors is generally more disorganized, potentiating migration of the material through poorly developed vessels beyond the tumor itself [54]. Finally, a significant complication of uterine fibroid embolization is post-embolization syndrome (PES), which is often seen when small particles (e.g., <500 um) are used and thought to result from microspheres reaching arterioles in close proximity to the fibroid [55,56]. As degradable microspheres shrink and likely advance further downstream within the tumor vascular bed, the risk of this complication may be increased. The complicated nature of diseased tissue combined with the novelty of degradable microspheres questions the applicability of the healthy models used in the papers under review as preclinical evaluations prior to clinical use. 

Embolization is performed clinically for several different indications not covered by the models explored in this review, such as arteriovenous malformation and hepatocellular carcinoma [6]. It should be noted these indications have a unique set of inherent risks, perhaps the most concerning of which is bypass of the target tissue with shunting to vital organs. The vascular networks of these pathologies, and hypervascular tumors in general, is highly variable and poorly organized, potentially enabling passage of microspheres to the systemic circulation. This may be of extreme clinical importance, especially with degradable materials, which are designed to decrease in size over time; the pressure of the pulsatile flow, coupled with the decreased size and altered compressibility of partially degraded microspheres may push the embolics further downstream. Several groups report pulmonary emboli when particles <10 µm are utilized [57], and radioactive Y-90 microspheres are designed to limit pulmonary radiation below a given threshold, indicating migration beyond target tissue is an acceptable event associated with the use of these products. Although the kidney and uterine models discussed above are clinically relevant, they do not address this important issue and therefore the commentary made in relation to migration does not translate to all indications.

## 5. Conclusions

This review identified five state of the art materials that have been investigated for use as degradable microspheres for TAE. In considering the FDA’s list of special controls for the preclinical evaluation of bland embolic microspheres, it is evident there are significant gaps in the current understanding of the safety, efficacy, and performance of these materials. More importantly, in reviewing this body of literature, it appears there is little consensus regarding the ideal characteristics of degradable microspheres. Specifically, ideal degradation timeframes, recanalization rates, and host responses (e.g., fibrotic ingrowth) have not been identified, making it difficult to establish precise design controls for the development of these products. Furthermore, the degradability of these microspheres present new and complex risks that have not been previously considered, the most important of which may be migration. As degradable microspheres shrink in size, they may (i) potentially advance beyond the intended level of occlusion resulting in more profound ischemia and necrosis and/or, (ii) reflux into adjacent vessels, resulting in non-target embolization and/or (iii) through capillaries (i.e., shunting) leading to unintended injury of next level organs such as the lung or brain. In the future, degradable embolic materials may be engineered so as to permit repeat embolization procedures with radioactive embolic particles, drug-eluting particles, and bland embolic agents. Doing so will permit physicians to achieve levels of occlusion on repeat procedures, and as a consequence may enhance the overall safety, efficacy, and performance of TAE procedures.

## Figures and Tables

**Table 1:** Materials reviewed and generalized search strategy parameters for PubMed and Web of Science.

Material Type	Acronym (If Applicable)	Standard Search Parameters
Poly(lactic-co-glycolic acid)	PLGA	“Material Type” ** AND “Microsphere”
PLGA-Polyethylene Glycol-PLGA	PLGA-PEG-PLGA	“Material Type” AND “Embolization”
Carboxymethylcellulose	CMC	“Material Type” AND “Occlusion”
Chitin		“Material Type” AND “Arterial”
Hydroxyethyl acrylate	HEA	“Material Type” AND “Radiology”
Albumin *		“Material Type” AND “Bead”
Gelatin		“Material Type” AND “Resorbable”
Pluronic F127		“Material Type” AND “Bioresorbable”
Polyvinyl alcohol	PVA	“Material Type” AND “Degradable”
Starch		“Material Type” AND “Bioabsorbable”

* “Albumin” + “arterial” was excluded due to the arterial presence of albumin; ** Note: The words ‘material type’ was replaced in each search by a given material of interest from the left hand column. Each material type was fully searched as per the search parameters in Table 1.

**Table 2:** Initial Returned Searches based on Table 1, with Articles Meeting Inclusion Criteria.

Material Type	Initial Returned Searches	Articles Meeting Inclusion Criteria	Article Title
PLGA	1662	1	A Preclinical Study of the Safety and Efficacy of OcclusinTM 500 Artificial Embolization Device in Sheep
PLGA-PEG-PLGA	985	2	A Novel Resorbable Embolization Microsphere for Transient Uterine Artery Occlusion: A Comparative Study with Trisacryl-Gelatin Microspheres in the Sheep Model
			Targeting and Recanalization after Embolization with Calibrated Resorbable Microspheres versus Hand-cut Gelatin Sponge Particles in a Porcine Kidney Model
CMC	417	1	Calibrated Bioresorbable Microspheres: A Preliminary Study on the Level of Occlusion and Arterial Distribution in a Rabbit Kidney Model
Chitin	585	1	Chitin-based Embolic Materials in the Renal Artery of Rabbits: Pathologic Evaluation of an Absorbable Particulate Agent
Hydroxyethyl acrylate	65	1	Transcatheter embolization using degradable crosslinked hydrogels
PVA	2014	0	
Albumin	6751	0	
Gelatin	2347	0	
Pluronic F127	41	0	
Starch	2083	0	
**TOTAL**	**16,950**	**6**	

**Table 3:** Pre-clinical safety summary for hydrolysis mediated degradable PLGA microspheres.

Authors and Year of Publication	Study Model & Duration	Test Material Information.	Ease of Use	Time to Complete Degradation of Test Material	Recanalization	Acute Complications (Vessel Rupture/Perforation)	Local and Systemic Foreign Body Reactions	Embolization Effectiveness.	Device Migration
Owen et al. (2012)	Uterine Artery Sheep Model 32 Suffolk cross sheep (Mean weight ca. 60 kg) Study duration: 12 months. Cohorts at 1, 3, 6 and 12 months. Control material: Embosphere 300–500 μm	PLGA 150–212 μm No particle size distribution analysis is provided.	UA selectively catheterized with either 2.3F Rapid Transit or 2.3F Prowler (Cordis Corporation). Fluoroscopic time to achieve stasis was comparable for the test article (8.9 ± 2.7 min) and Embosphere (8.1 ± 3.6 min) Suspensions in “a solution of normal saline and contrast medium’ were noted as being “easily delivered to target vasculature” using a 2.3F catheter.	Test material still present at 1 and 3 months. By 6 months the authors state that no residual material was observed, but occlusion remained persistent due to the presence of fibrous connective tissue.	3/4 animals treated with test article showed recanalization at 12 months. Recanalized vessels showed normal luminal architecture “histologically indistinguishable from the untreated contralateral vessel” No recanalization in Embosphere cohorts.	Vessel rupture not assessed. None reported.	Standard hematology and clinical chemistry parameters we performed prior to procedures, at 1, 7, 14 days, and 1, 3, 6, 12 months. No differences reported between test and control. At 1 month, fibrous connective tissue observed around test material, fully occludes treated vessels by 3 months and persists at 6 months. Vessels treated with test material were fully recanalized at 12 months and had similar architecture to untreated vessels. Microspheres of both types were embedded in a thin collagen matrix with small numbers of macrophages and occasional giant cells present. Yet inflammation was not a significant feature of the reaction to either type of microsphere.	Determined as being equivalent to Embosphere up to at least 6 months.	Not directly addressed. Histology showed test articles present in all 12 (100%) treated uterine arteries and in 1 untreated uterine artery, but not in vaginal, ovarian, or vesical vasculatures of any animal. Control articles were detected in all 16 (100%) treated uterine arteries, 6 (40%) untreated uterine arteries, as well in the vaginal vasculature of 10 (63%) animals, ovarian vasculature of 2 (13%), and Vesical vesicle vasculature of 1 (6%) animal.

**Table 4:** Pre-clinical safety summary for hydrolysis mediated degradable PEG-PLGA-PEG microspheres.

Authors and Year of Publication	Study Model & Duration	Test Material Information.	Ease of Use	Time to Complete Degradation of Test Material	Recanalization	Acute Complications (Vessel Rupture/Perforation)	Local and Systemic Foreign Body Reactions	Embolization Effectiveness.	Device Migration
Maeda et al. (2013)	Porcine Kidney Model. Study duration: 7 days. Control material: gelatin sponge particle (GSP) approx. 1 mm^3^ 9 Minipigs (Mean weight 34.9 kg ± 2.1 kg) Groups comprised two pigs (i.e., four kidneys per group)	PEG-PLGA-PEG 300–500 μm 500–700 μm 700–900 μm Size distributions determined by sieving only. No particle size distribution analysis is provided. 1:2 ratio of saline/contrast for test article with pure contrast for the control article	A 4-F cobra catheter was utilized for the embolization procedures. The mean volume of injected material per kidney was 0.48 mL ± 0.17, 0.24 mL ± 0.11, 0.24 mL ± 0.12 for REM of 300–500 μm, 500–700 μm, and 700–900 μm. Mean volume of control article injected was 1.2 mL ± 0.2 Authors note, “none of the products clogged in the catheter.”	Proposed as 24 h based on tests in PBS. At day 7 the test material was not visible, no fragments of materials were observed in histological slides/analysis. GSP was still present at day 7, though partly degraded. Its presence was associated with foreign body inflammation.	Assessed at 10 min and 7 days using angiography. Large variations due to methodology acknowledged. Assessed at 10 min and 7 days using histological analysis. At day 0 test materials were washed out during processing limiting analysis. Test article showed fully patent vessel lumens after 7 days Recanalization varied based on size of test material: 700–900 μm demonstrated complete recanalization 300–500 μm and 500–700 μm demonstrated partial recanalization.	Numerous patchy arterial lesions, including myointimal proliferation, medial concentric thickening, adventitial fibrosis, and fibrinoid necrosis of the arterial wall, were focally observed. No excessive pain or abnormal behavior reported	Local histological analysis provided. Hematoxylin-eosin-saffron stain used. GSP (control) had eosinophilic or slightly basophilic appearance at day 7 and partly degraded. Presence associated with foreign body inflammation (macrophages, lymphocytes and fibrocytes). Test materials were washed out during histological processing limiting analysis for day 0. At day 7, test material was not visible, no fragments of materials were observed in histological slides/analysis. Fully patent lumen visible on histology.	Recanalization demonstrated on angiography No gross histology to examine presence of long term necrosis	Not addressed.
Verret et al. (2014)	Uterine Artery Sheep Model 6 adult Préalpes Sheep. (Mean weight 54 kg) (Mean age 48 ± 22 months) Study duration: 7 days. Control material: Embosphere 500–700 μm	PEG-PLGA-PEG 500–700 μm Size distributions determined by sieving only. No particle size distribution analysis is provided. 2:1 ration of saline/contrast for test material and 4:5 ratio for control article	Selective embolization of both internal iliac arteries achieved using a 5F “cobra-type” catheter. Superselective embolization of both UAs performed with a 2.7F microcatheter. Mean volume of test material injected per uterine artery was 1.0 mL ± 0.5. Mean volume of control was 1.6 mL ± 0.9 No difference in injectability noted between control and test materials.	Proposed as 24hr based on tests in PBS. At day 7 the test material was not visible, no fragments of materials were observed in histological slides/analysis.	Presence or absence of recanalization assessed based on (i) the presence or absence of vascular lumen with (ii) red blood cells or plasma in the occluded vessel. For test article “complete recanalization rapidly obtained” and fully patent on angiography at 7 days.	Vessel rupture not assessed. None reported.	Local histological analysis provided. Gross examination showed ischemic damage to endometrium and myometrium for test and control uteri. Hematoxylin-eosin-saffron stain used. No test materials or inflammatory response observed at 7 days for test article. Control material showed evidence of recanalization, and was surrounded by macrophages, neutrophils and foreign body giant cells.	Gross examination showed ischemic damage to endometrium and myometrium for test and control uteri. The authors suggest that for the test article, full UA recanalization and absence of parenchymal defects were associated with low endometrial alterations.	Not addressed.

**Table 5:** Pre-clinical safety summary for hydrolysis mediated degradable CMC microspheres.

Authors and Year of Publication	Study Model & Duration	Test Material Information.	Ease of Use	Time to Complete Degradation of Test Material	Recanalization	Acute Complications (Vessel Rupture/Perforation)	Local and Systemic Foreign Body Reactions	Embolization Effectiveness.	Device Migration
Weng et al. (2013)	Renal Artery Rabbit Model 11 New Zealand white rabbits (Weight range 4–5 kg) 3 rabbits (group 1) received partial occlusion with BRMS-I (3, 15, and 25 mg of microspheres) To test the level of occlusion, 4 (group 2) received total occlusion with BRMS-I (10 mg/mL), and 4 more (group 3) rabbits received complete occlusion with BRMS-II Study duration: 15 min Tris-acryl gelatin microspheres (TGMS) (100–300 μm) were used as a control	2 test articles: BRMS-I and BRMS-II 2% (w/v) oxidized carboxymethylcellulose and 2% (w/v) carboxymethyl chitosan 10% oxidized carboxymethylcellulose was used in BRMS-I and 25% oxidized carboxymethylcellulose was used in BRMS-II 100–300 μm Average diameter of the microspheres was 250 μm ± 50 for BRMS-I, and 255 μm ± 45 for BRMS-II Concentration of microsphere suspension used was 1 mg/mL and 5 mg/mL for group 1, and 10 mg/mL for groups 2 and 3 all in a 5:5 saline:contrast solution	RA selectively catheterized a 4-F Cobra catheter inside which a 2.8-F microcatheter was placed Injection was “easily performed without any clogging or clumping” BRMS were deemed to be less “sticky” than TGMS BRMS-I required 8.7 mL ± 3.5 to achieve stasis and BRMS-II required 6.3 mL ± 0.8 Fluoroscopic time to achieve the endpoint was 4.5 min ± 1.6 for BRMS-I and 3.8 min ± 0.74	Not Addressed	Not addressed	Not addressed	Not addressed	Determined to achieve the desired goal of embolization similar to commercially available TGMS Mean diameter of occluded vessels found to be 197 μm ± 23 for BRMS-I, 219 μm ±36 for BRMS-II and 158 μm ±21 for TGMS	Not addressed

**Table 6:** Pre-clinical safety summary for hydrolysis mediated degradable Chitin microspheres.

Authors and Year of Publication	Study Model & Duration	Test Material Information.	Ease of Use	Time to Complete Degradation of Test Material	Recanalization	Acute Complications (Vessel Rupture/Perforation)	Local and Systemic Foreign Body Reactions	Embolization Effectiveness.	Device Migration
Kwak et al. (2005)	Renal Artery Rabbit Model 36 New Zealand white rabbits (weight range 2.0–3.5 kg) Study duration: 32 weeks. Cohorts at 1 and 3 days, and 1, 2, 4, 8, 16, 24, and 32 weeks Control material: PVA 150–250 μm	Chitin particles, 99% deacetylated chitosan particles, and 75% deacetylated chitosan microspheres 150–250 μm The length of chitin particles, chitosan particles, and chitosan microspheres was 335 μm ± 56.8, 466 μm ± 100.2, and 271 μm ± 37.2, respectively	RA selectively catheterized with 4-F angiography cobra catheter. No fluoroscopic time to achieve stasis given All four materials were noted as being “easily injected through the catheter without causing any blockage”	All embolic materials maintained their shape until week 8 Chitin particles showed fragmentation and absorption at week 24, absorbed completely at week 32 Chitosan particles showed fragmentation and absorption at week 16, absorbed completely by week 24 Chitosan microspheres showed degradation and absorptionat week 24, absorbed completely by week 32	Severe proliferations of the blood vessels by the retroperitoneal fat around the embolized kidney were observed from day 1 to week 1 for PVA, chitin and chitosan microspheres and from day 3 to week 2 for chitosan particles. Formation of capillaries were observed most frequently with PVA particles, followed by chitosan particles and chitosan microspheres	The degree of vascular injuries was moderately reactive with PVA particles and chitosan microspheres and substantially reactive with chitin particles No hemorrhage or extravasation for any of the embolic materials.	Giant cell reaction appeared prominently 1–2 weeks after embolization, and lasted until week 32. The degree of reaction was lowest with chitosan microspheres. As a whole, there was no substantial difference in gross observations among the four groups.	Chitosan microspheres were determined to be potential embolic agents as they block the blood vessels more compactly and with a lower rate of capillary formation than PVA particles.	Not addressed.

**Table 7:** Pre-clinical safety summary for hydrolysis mediated degradable HEA microspheres.

Authors and Year of Publication	Study Model & Duration	Test Material Information.	Ease of Use	Time to Complete Degradation of Test Material	Recanalization	Acute Complications (Vessel Rupture/Perforation)	Local and Systemic Foreign Body Reactions	Embolization Effectiveness.	Device Migration
Schwarz et al. (2003)	Renal Artery Canine Model 5 Beagles (Weight range 10–15 kg) 3 Kidneys were embolized with HEA Median auricular artery occlusion model 5 New Zealand rabbits (Weight range 2.5–3.5 kg) 4 central auricular arteries were embolized with HEA microspheres Study duration: 3 weeks. Monitored immediate and weekly through catheter angiography Control material: EmboGold microspheres 300–500 μm	Hydroxyethyl acrylate 300–500 μm No particle size distribution analysis is provided.	RA selectively catheterized with either 4-F or 5-F catheters. Catheterization of central artery of rabbit ears was performed with radiopaque catheters No comment made on ease of use	At 3 weeks, microspheres (sometimes intact but encapsulated, most often in various stages of degradation and phagocytosis) could be detected Occlusion lasted for the critical period at risk for recanalization, typically 10–14 days	Renal arterial occlusions that persisted at 1 week were recanalized at 3 weeks Experiments performed in the rabbit central auricular arterial model showed that HEA microspheres led to occlusions that persisted at 1 week but that recanalized at 2 weeks No recanalization in EmboGold cohorts.	Vessel rupture not assessed. None reported.	Only a residual inflammatory reaction and some neointimal thickening could be observed as a witness to the previous presence of these degradable microspheres	Determined as being potentially effective up to 2 weeks	Not addressed.
